# Missed opportunities for initiation of treatment and control of hypertension among older adults in India

**DOI:** 10.1016/j.pmedr.2022.102057

**Published:** 2022-11-15

**Authors:** Mrigesh Bhatia, Manish Kumar, Priyanka Dixit, Laxmi Kant Dwivedi

**Affiliations:** a Dept. of Health Policy, London School of Economics, London; b International Institute for Population Sciences, Mumbai, India; c Tata Institute of Social Sciences, Mumbai, India

**Keywords:** Hypertension, Missed opportunities, Treatment and control, Healthcare facilities, Access, Older adults, India

## Abstract

Hypertension (HT) is a major public health problem globally. The unacceptably low treatment and control rates are a major concern for policy makers as they contribute to avoidable mortality and morbidity. This study quantifies the prevalence and the determinants of missed opportunities for the treatment and control of HT in older adults in India. The study utilized data from the Longitudinal Aging Study in India (LASI), a population-based national representative survey of 62,416 individuals aged over 45 years. Our findings suggest that the prevalence of missed opportunities for the treatment and control of HT was 29.9 % and 16.4 % respectively. Overall, more than 60 % of all missed opportunities were in the private sector, and 75 % were in outpatient consultations. Education, working status, diabetes, stroke, physical activities, smoking, monthly per capita consumption expenditure (MPCE) quintiles were positively associated with missed opportunities for both treatment and control of HT. Rural residents, individuals with no comorbidities, and those belonging to lower MPCE quintiles were positively associated with missed opportunities for treatment. This association was inverse in the case of missed opportunities for the control of HT. Significant missed opportunities exist with respect to the treatment and control of HT. We discuss the reasons behind low treatment and control rates, including failure to initiate treatment, suboptimal compliance, and lack of follow-up, in the context of significant financial barriers to access to health services and availability of free anti-hypertensive drugs in India.

## 1 Introduction

Hypertension (HT) is a major public health problem that contributes to about 10.4 million deaths and 218 million DALYs worldwide (Stanaway et al., 2018). There are considerable global inequalities and huge variations across countries in the treatment and control of HT. These inequalities have continued to increase over the years (Mills et al., 2016). Over 80 % of all hypertensives in the world live in low- and middle-income countries (Zhou et al., 2021). In South Asia, HT is the third leading cause of death and disability (Lim et al., 2012).

A major concern in low- and middle-income countries is that a significant proportion of hypertensives are not aware of their hypertensive status (Lloyd-Sherlock et al., 2014). In absence of diagnosis, a substantial gap remains in the treatment and control of HT (Bhatia et al., 2021, Mohanty et al., 2021, Yang et al., 2016, Chow et al., 2013, Neupane et al., 2014). Globally, more than half of all hypertensives do not receive the necessary treatment (Zhou et al., 2021). A recent study estimated the treatment rate in the western world to be at 80 %. Globally, the rate varies from over 70 % in countries like the US, Canada, Portugal, and Iceland to less than 20 % in some Sub-Saharan African countries (Factsheet, 2021). HT control rates, on the other hand, are around 20–25 % globally and less than 10 % in some countries of Sub-Saharan Africa and Asia (Zhou et al., 2021). In India, over 200 million adults are estimated to have HT (Gupta et al., 2019), which contributes to 8 % of the total disability adjusted life years (DALYs) lost (Results, 2019). A study has shown that almost 40 % of hypertensive older adults are not aware of their condition and that only 10 % of all hypertensive adults over 45 years have their HT under control in India (Bhatia et al., 2021). With low treatment and control rates, India is projected to experience further rise in deaths and DALYs due to HT (Gupta and Gupta, 2017, Gupta et al., 2018).

Various studies have concluded that long-term uncontrolled and untreated HT significantly increases the risk of cardiovascular disease and mortality (Gu et al., 2010, Briganti et al., 2003, Pereira et al., 2009, Gaziano, 2011, Chobanian et al., 2003), puts undue pressure on the health systems by increasing the health care costs (Baruch, 2004), and results in financial hardship to households (Kastor and Mohanty, 2018). To meet the national and global targets, policy makers need to identify and implement effective low-cost strategies for the treatment and control of HT. One potential strategy could be to minimise the missed opportunities (MO) for the treatment and control of HT when hypertensive individuals avail public or private health facilities.

The concept of MO has been applied to health care in various clinical settings, screening of communicable and non-communicable diseases, implementation of vaccination programmes, and in the targeting of specific groups (Chihota et al., 2015, Schmittdiel et al., 2011, Abdulraheem et al., 2011, Jani et al., 2008, Creswell et al., 2011, Sheppard et al., 2014). More recently, this concept has been applied in the context of HT, with the suggestion that a substantial proportion of individuals remain undiagnosed despite having utilized healthcare facilities in the preceding year (Hashmi et al., 2016, Mohanty et al., 2021, Maurer and Ramos, 2015). Although missed opportunity has been explored with respect to the diagnosis of HT, to the best of our knowledge, our paper is one of the first ones to apply this concept in the context of treatment and control of HT in a low- and middle-income country. The present study aims to examine the prevalence and determinants of MO for treatment and control of HT among individuals aged 45 years or older in India.

## 2 Methods

### 2.1 Data source

We utilized data from the first wave of the Longitudinal Ageing Study in India (LASI, 2017–18), a nationally-representative survey that provides vital information about the social, physical, and mental well-being of older adults aged 45 years and over and their spouses across all 30 states (except Sikkim) and 6 union territories (UTs) in India. The Indian Council of Medical Research extended the ethical approval for conducting this survey. Our final sample consists of 62,416 older adults after excluding 3146 individuals with missing information on either outcome or explanatory variables.

### 2.2 Survey design

The LASI survey adopted a multistage stratified area probability cluster sampling design, stratified by urban and rural areas, with a goal of selecting representative sample in each stage of sample selection. Within each state, a four-stage sampling design in urban and a three-stage sampling design in rural area was adopted. A detailed description of the sampling design, survey questionnaires, fieldwork, and data collection is provided elsewhere (Mohanty et al., 2021). The response rates for the household and individual interviews were 93 % and 95 %, respectively.

### 2.3 Outcomes

The blood pressure was measured thrice with a one-minute gap using an electronic monitor (Omron model HEM-7121). Our study averaged the last two readings of the Systolic Blood Pressure (SBP) and Diastolic Blood Pressure (DBP). Based on the guidelines suggested by seventh report of the Joint National Committee on prevention, detection, evaluation, and treatment of high blood pressure (JNC-7), an individual was considered hypertensive if his/her average SBP was ≥ 140 mmHg or/and the average DBP was ≥ 90 mmHg or if the individual was using any antihypertensive medication at the time of the survey (Chobanian et al., 2003).

The outcome variables for the present study were MO for the treatment and control of HT. The prevalence of missed opportunity for the treatment of HT was defined as the proportion of untreated individuals among the self-reported hypertensive cases who reported that they are not hypertensive but in biometric measurement were found to be hypertensive, and had visited a public or private health facility in the preceding 12 months. On the other hand, the prevalence of MO for the control of HT was defined as the proportion of individuals with uncontrolled HT, among all the hypertensive cases, who had been consuming antihypertensive drugs at the time of the survey and had visited a public or private health facility in the preceding 12 months (See Fig. 1).

### 2.4 Covariates

The analysis included a set of individual, health, lifestyle, and household factors to assess the determinants of MO for the treatment and control of HT. The individual factors consisted of sex (male, female), age group (45–54 years, 55–64 years, 65–74 years, 75 + years), educational status (no education, primary, secondary, higher), working status (never worked, currently working, not currently working), marital status (currently married, widowed, divorced/separated/deserted), and health insurance (yes, no). The health factors included physical and functional health. Three self-reported chronic conditions, namely diabetes, stroke, and arthritis were also included. The functional health of the participants was estimated by evaluating the limitations in performing the basic activities of daily living (ADLs) and instrumental activities of daily living (IADLs). Participants with at least one difficulty in executing basic or instrumental ADLs were categorized as “Yes.” LASI also collected information on the performance of physical activities (moderate and vigorous) and the consumption of tobacco and alcohol among older adults. We included household factors too such as monthly per capita consumption expenditure (MPCE) quintile (poorest, poorer, middle, richer, richest), religion (Hindu, Muslim, Christian, others), caste (Scheduled Caste, Scheduled Tribe, Other Backward Class (OBC), others), and place of residence (rural, urban).

### 2.5 Statistical analysis

We calculated the prevalence rates of the outcome variables by public and private health facilities. We constructed funnel plots to observe the variations in MO for the treatment and control of HT across the states. The national average of MO for the treatment and control for HT was used as the baseline reference in these plots. We created the 95 % and 99 % confidence bands in the funnel plots. The sample was described by using descriptive analysis. A bivariate analysis was conducted by using the Chi-square test to examine the potential associations of various individual, health, lifestyle, and health factors with the outcome variables. We used multivariable logistic regression to assess the association of MO for the treatment and control of HT with various covariates mentioned above.

## 3 Results

Table S1 presents the sample characteristics and HT prevalence according to various individual, health, lifestyle, and household-related factors. A total of 26,597 (40.4 %-weighted) out of 62,416 older adults had HT (Supplementary material – Table S1). Table 1 presents the prevalence of MO for the treatment and control of HT by the type of health facility utilized during 12 months preceding the survey. Overall, the prevalence of MO for the treatment and control of HT among hypertensive individuals was 29.9 % and 16.4 % respectively. The MO for both treatment and control was higher in private than in the public health facilities. In the case of treatment, the MO was significantly higher among males, in the age group 45–54 years, uneducated, with no health insurance, and those who had no comorbidities. By contrast, in the case of control of HT, the prevalence of MO was significantly higher among females, who were educated, with co-morbidities, had difficulty performing activities of daily living, and were physically inactive.

**Table 1:** Missed opportunities of treatment and control for hypertension among older adults aged 45 years and over, by various background characteristics, LASI, 2017–18.

Background characteristics	Treatment^1^			Control^2^		
	Total %	Public facility %	Private facility %	Total	Public facility	Private facility
**Overall**	**29.9**	**11.2**	**22.9**	**16.4**	**5.6**	**13.1**

Individual factors						
Sex						
Male	32.3	12.2	24.5	14.7	5.0	11.9
Female	28.1	10.4	21.5	17.7	6.1	14.0
Age groups						
45–54	30.9	11.1	23.8	12.5	4.6	9.9
55–64	30.2	11.0	23.4	16.5	5.4	13.7
65–74	29.6	11.5	22.8	18.7	6.6	14.4
75+	28.1	11.0	20.0	18.7	5.6	15.1
Education level						
No education	33.8	12.9	25.5	15.4	5.9	11.9
Primary	31.3	12.1	23.5	17.3	6.3	13.6
Secondary	25.3	8.8	19.8	18.4	5.2	15.2
Higher	18.9	5.7	15.6	15.4	3.2	13.5
Working status						
Never worked	22.2	7.9	16.9	19.2	5.9	15.7
Currently working	36.3	12.8	28.3	11.3	3.8	9.1
Not currently working	29.1	12.2	21.6	20.0	7.4	15.7
Marital Status						
Currently married	30.3	10.6	23.8	15.8	5.0	13.1
Widowed	28.5	12.2	20.4	17.8	6.8	13.3
D/S/D/Others	35.8	15.2	26.0	15.9	8.4	9.2
Health insurance						
No	30.2	10.4	23.7	15.9	5.1	12.9
Yes	28.8	13.9	19.8	18.0	7.3	13.7

Health factors						
Diabetes						
No	33.8	12.7	25.8	13.2	4.7	10.4
Yes	15.3	5.4	11.9	29.4	9.3	24.2
Stroke						
No	30.4	11.3	23.3	15.8	5.2	12.6
Yes	13.6	6.4	9.7	36.1	17.5	29.4
Arthritis						
No	30.4	11.1	23.4	15.8	5.3	12.7
Yes	25.8	12.0	18.0	21.0	7.9	16.3
Difficulty in ADL						
No	30.0	11.1	23.0	15.2	5.0	12.2
Yes	29.6	11.4	22.1	21.3	8.0	17.0
Difficulty in IADL						
No	29.5	10.7	22.7	14.7	4.7	11.9
Yes	30.6	11.8	23.1	18.9	6.9	14.9

Lifestyle factors						
Moderate activities						
Inactive	27.7	9.9	21.2	17.1	5.9	13.5
Active	31.4	12.0	24.0	15.9	5.4	12.8
Vigorous activities						
Inactive	28.4	10.8	21.5	17.7	6.2	14.0
Active	34.0	12.2	26.5	13.0	3.9	10.8
Smoking status						
Never	28.9	10.4	22.3	16.7	5.4	13.4
Former	30.4	12.7	22.5	24.5	11.1	20.8
Current	38.0	16.8	27.4	10.7	4.8	7.6
Chewing tobacco						
Never	27.8	10.2	21.1	17.1	5.6	13.8
Former	32.6	12.0	25.7	17.0	6.0	13.8
Current	38.5	15.2	29.7	13.2	5.3	10.1
Ever used alcohol						
No	28.6	10.4	22.0	17.0	5.7	13.5
Yes	37.2	15.2	27.6	13.1	5.1	10.8

Household factors						
MPCE quintile						
Poorest	34.9	14.5	24.7	11.8	5.1	8.3
Poorer	32.4	13.2	24.2	15.0	4.8	11.8
Middle	30.9	11.1	23.8	17.2	6.9	13.5
Richer	28.7	10.1	22.4	18.1	5.7	14.9
Richest	23.4	7.5	19.5	19.4	5.4	16.5
Religion						
Hindu	30.4	11.6	23.2	15.5	5.1	12.5
Muslim	27.9	9.5	22.5	20.1	8.1	14.9
Christian	22.7	12.3	12.0	17.4	6.6	13.1
Others	30.8	7.5	25.6	22.4	6.8	19.1
Caste						
Scheduled caste	34.1	15.3	25.4	14.8	6.1	10.7
Scheduled tribe	33.5	17.6	20.4	7.8	3.4	5.7
OBC	28.7	9.8	22.1	16.6	5.4	13.7
Others	28.4	9.1	23.1	19.4	6.1	15.8
Place of residence						
Rural	35.8	13.4	27.5	14.7	5.6	11.5
Urban	20.8	7.7	15.5	19.1	5.5	15.8

*Note.*^1^Among hypertensive individuals, all individuals those who were not taking treatment but visited the health facility in the last 12 months, ^2^Among the hypertensive cases, individuals consuming the antihypertensive drugs at the time of the survey and had visited the health facility in the last 12 months but had uncontrolled HT, D/S/D/Others-divorced, separated, or deserted, OBC- Other Backward Class, and MPCE- Monthly Per capita Consumption Expenditure.

Table 2 shows the MO for the treatment and control of HT by type of health care facility (public and private) and type of service (outpatient and inpatient). Overall, more than 60 % of all MO for the treatment and control were in the private sector, and 3 out of 4 MO occurred in outpatient consultations. The MO for the treatment of HT was greater in public facilities than in private facilities for both inpatient (39.5 vs 28.1 %) and outpatient (46.7 vs 38.3 %) visits. It is interesting to note that the MO for treatment decreased with increasing MPCE quintiles for both inpatient and outpatient visits in public as well as private facilities but increased in the case of control of HT. Table S2 provides MO for treatment and control by public and private facilities for individual States in India (Supplementary material; Table S2).

**Table 2:** Missed opportunities of treatment and control of hypertension among older adults aged 45 years and over according to MPCE quintiles, by type of health facility and type of service (outpatient and inpatient), LASI, 2017–18.

	Missed opportunities for treatment											
	Public						Private					
	Inpatient^1^		Outpatient^2^		Total		Inpatient^1^		Outpatient^2^		Total	
	N	%	N	%	N	%	N	%	N	%	N	%
MPCE quintile												
Poorest	68	53.7	429	52.5	474	53.2	28	27.3	534	51.8	550	51.4
Poorer	66	44.9	435	53.2	475	51.8	55	33.1	628	41.5	659	41.6
Middle	70	39.2	403	47.3	454	47.2	68	35.0	696	40.8	726	40.8
Richer	55	34.9	387	42.0	422	41.6	98	33.1	769	37.4	812	37.0
Richest	65	25.9	316	36.6	355	35.3	129	20.3	736	27.6	804	27.9
**Total**	**324**	**39.5**	**1,970**	**46.7**	**2,180**	**46.2**	**378**	**28.1**	**3,363**	**38.3**	**3,551**	**38.2**

	Missed opportunities for Control											
MPCE quintile												
Poorest	26	18.0	187	20.2	198	19.7	27	24.6	226	19.9	244	20.3
Poorer	35	20.9	210	20.2	230	20.6	41	19.6	369	24.5	390	24.2
Middle	50	23.2	250	22.7	279	22.7	56	27.9	444	24.6	463	24.6
Richer	48	27.7	220	21.8	251	22.9	79	25.4	549	26.7	582	26.5
Richest	66	40.9	248	24.7	291	27.5	131	29.0	573	26.3	628	26.5
**Total**	**225**	**26.3**	**1,115**	**21.8**	**1,249**	**22.5**	**334**	**26.1**	**2,161**	**24.8**	**2,307**	**24.8**

Note. MPCE- Monthly Per capita Consumption Expenditure. ^1^are the individuals who were admitted as patient to a hospital/long-term care facility for at least one night in past 12 months. ^2^are the individuals who received healthcare or consultation from a healthcare provider (including home visits) in past 12 months.

**Fig. 2:**
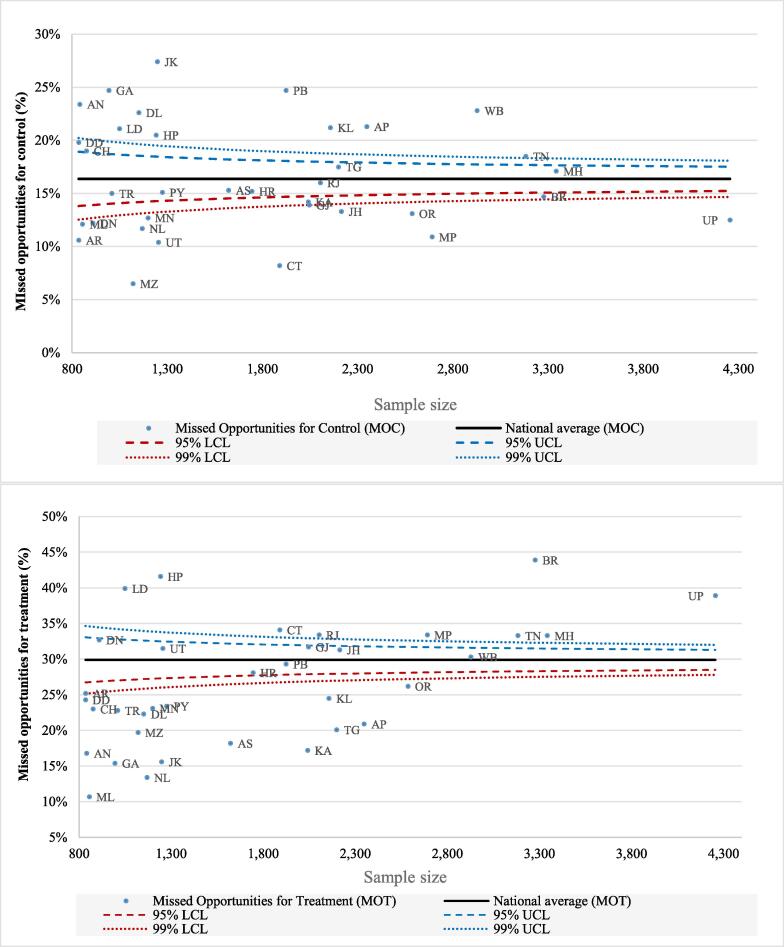
Funnel plots for the missed opportunities for treatment and control of hypertension, LASI 2017–18.

Fig. 2 shows the funnel plots of the prevalence of MO for the treatment and control of HT in different states of India. The prevalence rates in various states were compared with the national average that is represented by the solid black line parallel to the x-axis. Data points closer to the y-axis show states with smaller population size, while those away from the y-axis show states with larger population size. States above the national average show greater MO for the treatment and control of HT. The figures suggest that the prevalence of MO for the treatment of HT was higher in Uttar Pradesh, Bihar, Himachal Pradesh, and Chhattisgarh and lower in Kerala, Karnataka, and Jammu and Kashmir. The MO for the control of HT was higher in the states of Jammu and Kashmir, Goa, Punjab, and West Bengal and lower in Mizoram, Chhattisgarh, Uttarakhand, and Madhya Pradesh. Names of the states are provided in the Supplementary material (Table S3).

Table S4 presents the background characteristics of the individuals with controlled and uncontrolled HT (Supplementary Material, Table S4). As compared to individuals with uncontrolled HT, individuals with controlled HT were more likely to be younger, have higher level of education, physically active, in richest quintile, are urban resident, and less likely to have functional limitations and co-morbidities. Table 3 summarizes the results of the adjusted logistic regression for missed opportunities (MO) for the treatment and control of HT by the selected covariates. The results suggest that age and sex were not associated with MO for the treatment and control of HT. In terms of MPCE quintiles, individuals in the richest group were significantly less likely to miss the opportunity for treatment (OR: 0.70, 95 %CI: 0.59–0.83) than individuals in the poorest group, after controlling for all background characteristics. However, the MPCE quintiles were positively associated with the MO for the control of HT. Individuals with diabetes and stroke were significantly less likely to miss the opportunity for treatment but significantly more likely to miss the opportunity to control HT. Urban residents were significantly less likely to miss the opportunity for treatment (OR: 0.61, 95 %CI: 0.53–0.71) than rural residents. We did not find any association of marital status, physical activity, and functional health with the MO for the control of HT.

**Table 3:** Multivariable logistic regression results for missed opportunities for treatment and control of hypertension according to various background characteristics, LASI, 2017–18.

	Treatment^1^		Control^2^	
	OR	95 %CI	OR	95 %CI
Individual factors				
Sex				
Male	*Ref.*		*Ref.*	
Female	0.96	[0.85,1.09]	1.15	[0.98,1.35]
Age groups				
45–54	*Ref.*		*Ref.*	
55–64	0.97	[0.85,1.10]	1.15	[0.91,1.44]
65–74	1.02	[0.87,1.19]	1.17	[0.98,1.40]
75+	0.93	[0.76,1.12]	1.19	[0.94,1.51]
Education level				
No education	*Ref.*		*Ref.*	
Primary	0.99	[0.87,1.13]	1.05	[0.88,1.25]
Secondary	0.83*	[0.72,0.97]	1.03	[0.76,1.39]
Higher	0.63**	[0.47,0.84]	0.84	[0.60,1.16]
Currently working				
Never worked	*Ref.*		*Ref.*	
Currently working	1.65***	[1.41,1.92]	0.80*	[0.65,0.98]
Not currently working	1.34***	[1.14,1.57]	1.18	[0.95,1.47]
Marital status				
Currently married	*Ref.*		*Ref.*	
Widowed	0.94	[0.84,1.05]	0.98	[0.81,1.19]
D/S/D/Others	1.24	[0.94,1.63]	1.05	[0.73,1.51]
Health insurance				
No	*Ref.*		*Ref.*	
Yes	0.89	[0.79,1.01]	1.30***	[1.15,1.48]

Health factors				
Diabetes				
No	*Ref.*		*Ref.*	
Yes	0.46***	[0.39,0.53]	2.38***	[2.07,2.74]
Stroke				
No	*Ref.*		*Ref.*	
Yes	0.40***	[0.30,0.54]	2.27***	[1.62,3.19]
Arthritis				
No	*Ref.*		*Ref.*	
Yes	0.93	[0.79,1.09]	1.09	[0.79,1.48]
Difficulty in ADL				
No	*Ref.*		*Ref.*	
Yes	1.04	[0.89,1.20]	1.30*	[1.06,1.60]
Difficulty in IADL				
No	*Ref.*		*Ref.*	
Yes	1.06	[0.94,1.19]	1.04	[0.85,1.27]

Lifestyle factors				
Moderate activities				
Inactive	*Ref.*		*Ref.*	
Active	1.21***	[1.08,1.35]	1.03	[0.90,1.17]
Vigorous activities				
Inactive	*Ref.*		*Ref.*	
Active	0.97	[0.84,1.11]	0.96	[0.81,1.13]
Smoking status				
Never	*Ref.*		*Ref.*	
Former	0.97	[0.77,1.22]	1.56*	[1.06,2.30]
Current	1.16	[0.98,1.36]	0.73**	[0.58,0.92]
Chewing tobacco				
Never	*Ref.*		*Ref.*	
Former	1.12	[0.88,1.43]	0.87	[0.61,1.25]
Current	1.25***	[1.11,1.40]	0.86*	[0.75,1.00]
Ever used alcohol				
No	*Ref.*		*Ref.*	
Yes	1.16*	[1.00,1.35]	0.95	[0.79,1.14]

Household factors				
MPCE quintile				
Poorest	*Ref.*		*Ref.*	
Poorer	0.9	[0.78,1.03]	1.30**	[1.07,1.60]
Middle	0.89	[0.75,1.04]	1.49***	[1.20,1.84]
Richer	0.83**	[0.72,0.96]	1.44***	[1.18,1.77]
Richest	0.70***	[0.59,0.83]	1.53***	[1.24,1.89]
Religion				
Hindu	*Ref.*		*Ref.*	
Muslim	1.02	[0.81,1.27]	1.33**	[1.10,1.62]
Christian	0.69**	[0.53,0.90]	1.15	[0.88,1.52]
Others	1.06	[0.87,1.29]	1.61***	[1.26,2.07]
Caste				
Scheduled caste	*Ref.*		*Ref.*	
Scheduled tribe	0.83	[0.69,1.01]	0.56***	[0.42,0.73]
OBC	0.97	[0.84,1.12]	1.01	[0.82,1.25]
Others	1.09	[0.93,1.28]	1.20	[0.98,1.46]
Place of residence				
Rural	*Ref.*		*Ref.*	
Urban	0.61***	[0.53,0.71]	1.11	[0.94,1.32]

*Note.*^1^Among hypertensive individuals, all individuals those who were not taking treatment but visited the health facility in the last 12 months, ^2^Among the hypertensive cases, individuals consuming the antihypertensive drugs at the time of the survey and had visited the health facility in the last 12 months but had uncontrolled HT, D/S/D/Others-divorced, separated, or deserted, OBC- Other Backward Class, IADL- Instrumental activities of daily living, and MPCE- Monthly Per capita Consumption Expenditure.

*p ≤ 0.05, **p ≤ 0.01, ***p ≤ 0.001.

## 4 Discussion

Although a few studies have reported that a substantial proportion of individuals remain undiagnosed despite having utilized healthcare facilities in the preceding year (Hashmi et al., 2016, Mohanty et al., 2021, Maurer and Ramos, 2015), there are hardly any studies that have assessed the MO for the treatment and control of HT. For example, a previous study on the MO for the diagnosis of HT in India found that 23 % of the undiagnosed hypertensives had made a visit to either a public or a private facility during a period of one year preceding the survey (Mohanty et al., 2021). Similarly, a multi-country study based on SAGE data found that the missed opportunity for diagnosis varied from 12 % in Russia to 37 % in Ghana, with the MO being 33 % in India (Maurer and Ramos, 2015). Our study contributes to the limited empirical literature in the field by focussing on the MO for the treatment and control of HT among older adults in India. We found that the prevalence of the MO for the treatment and control of HT was 30 % and 16 %, respectively, among hypertensive adults aged 45 years and over. Overall, more than 60 % of all MO for both treatment and control were in the private sector, and 3 out of 4 MO occurred in outpatient consultations. Our findings suggest that a significant number of visits to health facilities are associated with MO for the initiation of treatment of HT and its control. A US study on the missed opportunity for uncontrolled HT concluded that of the 36 million Americans with uncontrolled HT, 72 % had seen a doctor at least twice in the previous year (Centers for Disease Control and Prevention (CDC), 2012).

The present study also unveils the potential determinants of the MO for the treatment and control of HT. The results obtained from our multivariable logistic analysis suggest that urban residents, individuals with comorbidities, and those belonging to higher MPCE quintiles were negatively associated with MO for the treatment but positively associated with MO for the control of HT. Our finding as to the missed opportunity being lower among people with chronic conditions like diabetes and stroke can be explained by the fact that being at high risk, such individuals are routinely screened for HT when visiting a health facility. A study has reported that individuals of a higher socioeconomic status are also more aware of their chronic conditions (Bhan et al., 2017). Our finding shows that people in the richest MPCE category had a lower likelihood of missing the opportunity for treatment may be explained by the higher awareness regarding treatment and control of HT in this group (Mohanty et al., 2021, Bhan et al., 2017). However, despite taking antihypertensive treatment, a greater proportion of individuals in the richest category failed to control their blood pressure than those in the poorest category. This may be attributed to lifestyle factors, including the consumption of an unhealthy diet and a higher consumption of cigarettes.

As the literature on the rate of conversion of MO into improved treatment rates is scarce, we performed a scenario analysis with conversion rates of 25 % to 100 %. Extrapolating our findings to India as a whole, we estimate that about 6 million visits are MO for treatment of HT over a one-year period, with a majority of the MO for treatment occurring among the poor, rural residents, and those with no comorbidities.

The fact that control of blood pressure was not achieved among the study respondents may be attributed to either inadequacy of the existing treatment regime or to non-compliance with the treatment regime (Burnier and Egan, 2019). Among those with uncontrolled HT, the existing treatment plan needs to be re-assessed so that either another anti-hypertensive drug is added or a higher level/new class of anti-hypertensive drug is prescribed. Non-compliance can arise from a number of factors, including lack of awareness about the importance of anti-hypertensive treatment, reluctance to take regular treatment due to being asymptomatic initially, poor health-seeking behaviour, barriers in accessing to health facilities, and affordability of anti-hypertensive drugs. Despite a clinical diagnosis, a substantial proportion of individuals find it challenging to follow the antihypertensive treatment or make appropriate lifestyle changes such as quitting smoking and eating a healthy diet (Centers for Disease Control and Prevention (CDC), 2010).

Literature suggests that financial barriers can be a significant concern since both the diagnosis of HT and the purchase of hypertensive drugs require out-of-pocket expenditure (OOPE) by a majority of the population in a country like India. The problem is exacerbated by the fact that diagnostic services, doctor consultations, or admissions in the private sector are all on a fee-for-service basis. There is evidence to suggest that high OOPE for health care contributes to impoverishment in India (Ghosh, 2011). A study in India found that expenditure on medicines can be as high as 70 % of the overall out-of-pocket payments on health (Garg and Karan, 2009), and that one in four adults in India do not take HT treatment daily (Sudharsanan et al., 2021). Given that only 23 % of hypertensives are insured, financial affordability both in terms of regular physician follow-up and purchase of drugs can be an important concern. Hence, removing financial barriers to access to hypertensive drugs would immensely help improve treatment and control rates of HT.

The Government of India’s Non-Communicable Diseases (NCD) target includes a 25 % reduction in the overall mortality from cardiovascular diseases and a 25 % relative reduction in raised blood pressure prevalence (Ministry of Health Family Welfare (MoHFW), 2017). The Government of India launched the National Programme for the Prevention and Control of Cancer, Diabetes, Cardiovascular Diseases and Stroke in 2010–11, and more recently, in 2017, the India HT Control Initiative (IHCI) to ensure continuum of care for hypertensive patients and provision of free anti-hypertensive drugs. By 2020, IHCI had been implemented across 31 districts in 6 states and had enrolled over 8 lakh beneficiaries (Kaur et al., 2021). In addition, both population-based screening (PBS) and opportunistic screening for HT are an integral part of these programmes and initiatives. However, only 4 million persons attended NCD clinics and were screened for HT in 2018 (Yang et al., 2016). Similarly, under PBS for diabetes, HT, and common cancers, only about 11 million individuals had been covered (NPCDCS, 2020). Although commendable, these efforts still appear to be in infancy as India needs to deal with an estimated 200 million hypertensives, a significant proportion of whom are undiagnosed and untreated and have uncontrolled HT.

The challenges involved in controlling HT and the need for regular follow-up were highlighted in a recent evaluation of IHCI sentinel sites of more than 21,000 patients, which found that nearly half of the patients (51 %) did not turn up for a scheduled follow-up visit^49^. Failure to initiate treatment, suboptimal compliance, and lack of follow-up are all responsible for the low treatment and control rates of HT in India, leading to unnecessary morbidity and mortality. A number of solutions can be explored at individual, provider and systems levels. Individuals could be made aware of their condition and of the benefits of taking regular medication. Providers could be trained to follow standard treatment protocols and evidence-based guidelines, prescribe anti-hypertensive drugs that are available on the drug list, take blood pressure measurements at every opportunity, ensure regular follow-up of patients, and implement appropriate referral pathways. Systems-level measures would be to ensure the availability of blood pressure monitoring facilities, minimise barriers to access to health facilities, and make anti-hypertensive drugs available for free. A systems approach would ensure better reporting and co-ordination at various levels of health facilities and between the public and the private sector.

The strength of this study is that, to the best of our knowledge, our paper is one of the first to quantifying the magnitude of MO for the treatment and control of HT from a low- and middle-income country, and thus makes an empirical contribution in this field. All the limitations of cross-sectional study apply to this work, including recall bias. In addition, as BP measurement was taken on the day of the survey, whereas questions on the utilisation of the health facility were asked for a one-year period preceding the survey, this could be a potential source for bias. Finally, given the complexities involved in controlling BP and the fact that it would be unrealistic to expect improvements in control rates from a single visit to a health facility, the scenario analysis was not performed for control rates. Further primary research may be undertaken to assess the effectiveness of reducing MO and the conversion rates in terms of improvement in treatment and control of HT across health facilities in the public and private sectors.

## 5 Conclusion

In spite of HT being a major public health problem, policy makers in low and middle income countries continue to face the challenge of improving low treatment and control rates of HT. Our findings suggest that significant MO exist with respect to the treatment and control of HT both in the public and the private health sector in India. Given the chronically underfunded health system in India, minimising MO, could be an important low-cost strategy in improving treatment and control rates of HT. In addition, improving access to health services and providing free anti-hypertensive drugs would be a step in the right direction towards meeting the national NCD targets and preventing avoidable morbidity and mortality due to cardio vascular diseases.

## Ethics

The Indian Council of Medical Research (ICMR) extended the necessary guidelines and ethics approval for undertaking the LASI survey. All methods were carried out in accordance with the relevant guidelines and regulations of the Indian Council of Medical Research (ICMR). The survey agencies that conducted the field survey for data collection had obtained prior informed consent from the respondents.

## Funding

This research has received funding from the LSE Covid Impact Fund for Research and Knowledge Exchange.

## Availability of data and materials

The study used secondary data, which is available in a private database and accessible on reasonable request at https://www.iipsindia.ac.in/content/lasi-wave-i.

## CRediT authorship contribution statement

**Mrigesh Bhatia:** Conceptualization, Writing – original draft, Funding acquisition, Supervision. **Manish Kumar:** Conceptualization, Writing – original draft, Software, Data curation, Formal analysis. **Priyanka Dixit:** Writing – original draft, Validation. **Laxmi Kant Dwivedi:** Conceptualization, Formal analysis, Validation, Supervision.

## Declaration of Competing Interest

The authors declare that they have no known competing financial interests or personal relationships that could have appeared to influence the work reported in this paper.

## Data Availability

Data will be made available on request.
